# Supplementary material for: A class I PI3K signalling network regulates primary cilia disassembly in normal physiology and disease
Source: Nat Commun. 2024 Aug 21;15:7181. doi: 10.1038/s41467-024-51354-1 (PMC11339396; doi:10.1038/s41467-024-51354-1)

A class I PI3K signalling network regulates primary cilia disassembly in normal physiology and disease

Sarah E. Conduit[1]*, Wayne Pearce[1], Amandeep Bhamra[2], Benoit Bilanges[1], Laura Bozal-Basterra[3,4], Lazaros C. Foukas[5], Mathias Cobbaut[6], Sandra D. Castillo[7], Mohammad Amin Danesh[1], Mahreen Adil[1], Arkaitz Carracedo[3,4,8,9,10], Mariona Graupera[4,7,11], Neil Q. McDonald[6,12], Peter J. Parker[13,14], Pedro R. Cutillas[15], Silvia Surinova[2], Bart Vanhaesebroeck[1]*

Supplementary Information

Supplementary Figure 1

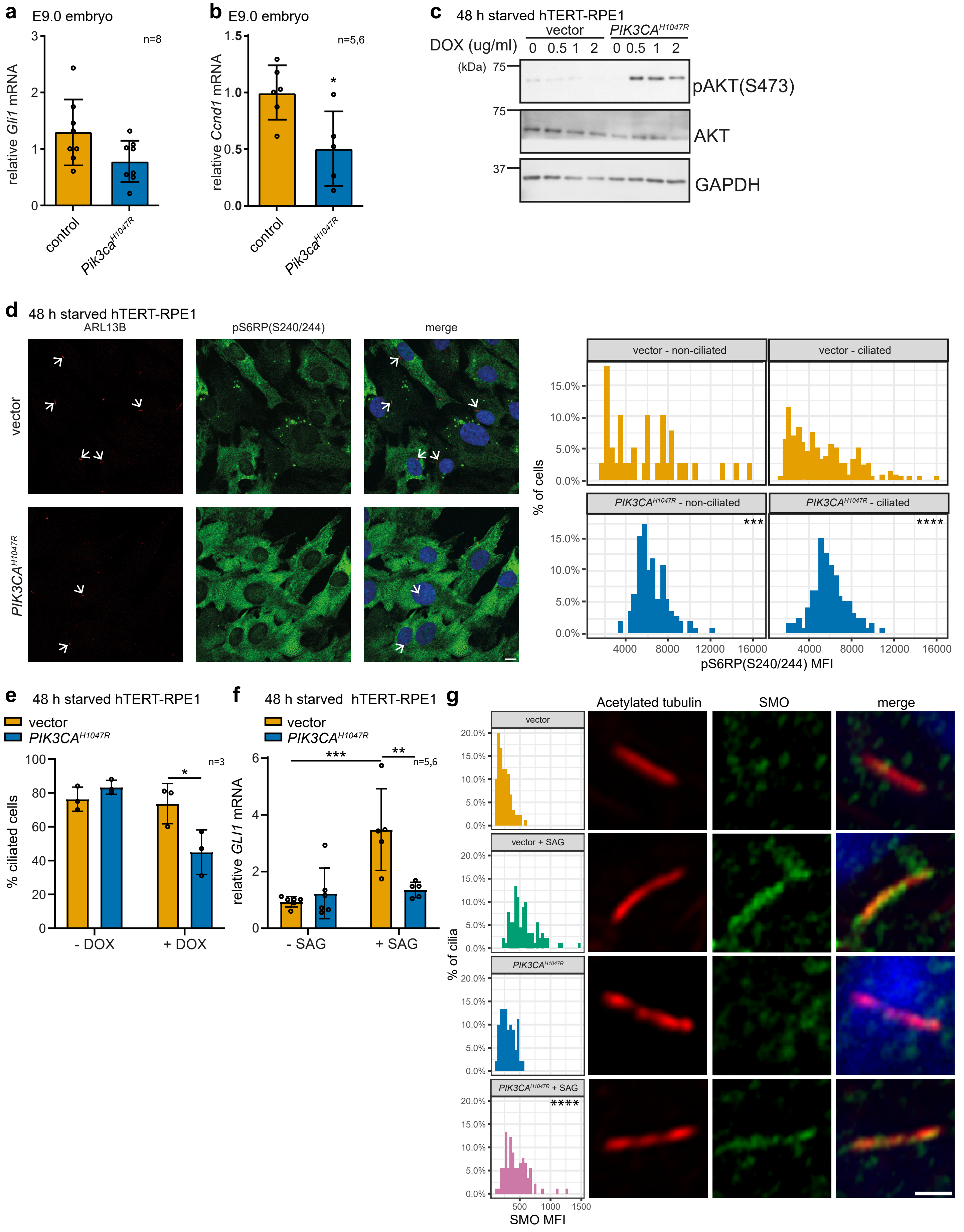

Supplementary Fig. 1 | Repression of Hedgehog and Wnt target genes in *Pik3ca*^{H1047R} embryos. a-b, E9 control and *Pik3ca*^{H1047R} embryos were lysed and (**a**) *Glli1* or (**b**) *Ccnd1* mRNA levels quantified relative to *Gapdh* by qRT-PCR, bars represent mean $\pm$ SD, (**a**) n=8, (**b**) n=5,6 mice of each genotype, * p<0.05 (Student's t-test, (**a**) p=0.0522, (**b**) p=0.0179). **c,** Vector and *PIK3CA*^{H1047R} hTERT-RPE1 cells were treated with 0, 0.5, 1 or 2 μg/ml doxycycline (DOX) for 48 h in serum free media, lysed and immunoblotted with pAKT(S473), AKT and GAPDH antibodies, blots representative of n=3 independent experiments. **d,** Vector and *PIK3CA*^{H1047R} hTERT-RPE1 cells were serum-starved for 48 h with 0.5 μg/ml doxycycline, fixed, stained with ARL13B and pS6RP(S240/244) antibodies and DAPI and imaged by confocal microscopy, arrows indicate cilia, bar indicates 10 μm. The pS6RP(S240/244) MFI was measured in non-ciliated and ciliated cells and presented as a histogram. n>220 cells per condition from 3 independent experiments, ***p=0.0001 (Kolmogorov-Smirnov test, p=1.334x10^{-4}) for non-ciliated vector vs *PIK3CA*^{H1047R} and ****p<0.0001 (Kolmogorov-Smirnov test p= 0.004556) for ciliated vector vs *PIK3CA*^{H1047R}. **e,** Vector and *PIK3CA*^{H1047R} hTERT-RPE1 cells were serum-starved for 48 h $\pm$ 0.5 μg/ml doxycycline (DOX), fixed, stained with antibodies to ARL13B or pericentrin and DAPI and the percentage of ciliated cells scored, bars indicate mean $\pm$ SD, 100 cells scored per condition per independent experiment, n=3 independent experiments, *p<0.05, (two-way ANOVA, interaction p=0.0132, row factor p=0.0066, column factor p=0.0904). **f,** Vector and *PIK3CA*^{H1047R} hTERT-RPE1 cells were serum-starved for 48 h with 0.5 μg/ml doxycycline and treated $\pm$ 400 nM SAG for the final 24 h, lysed and *GLI1* mRNA levels quantified by qRT-PCR relative to *GAPDH*, bars indicate mean $\pm$ SD, n=5,6 independent experiments, **p<0.01, ***p<0.001 (two-way ANOVA interaction, p=0.0036, row factor p=0.0015, column factor p=0.0196). **g,** Vector and *PIK3CA*^{H1047R}-transduced hTERT-RPE1 cells were serum-starved for 48 h with 0.5 μg/ml doxycycline +/- 24 h 200 nM SAG, fixed, stained with antibodies to acetylated tubulin or SMO and DAPI and imaged by confocal microscopy, bar indicates 1 μm. The ciliary SMO MFI was measured and presented as a histogram. n=90 cells per condition from 3 independent experiments ****p<0.0001 (Kolmogorov-Smirnov test for *PIK3CA*^{H1047R} + SAG vs Vector + SAG, p= 4.610x10^{-5}). Source data are provided as a Source Data file.

Supplementary Figure 2

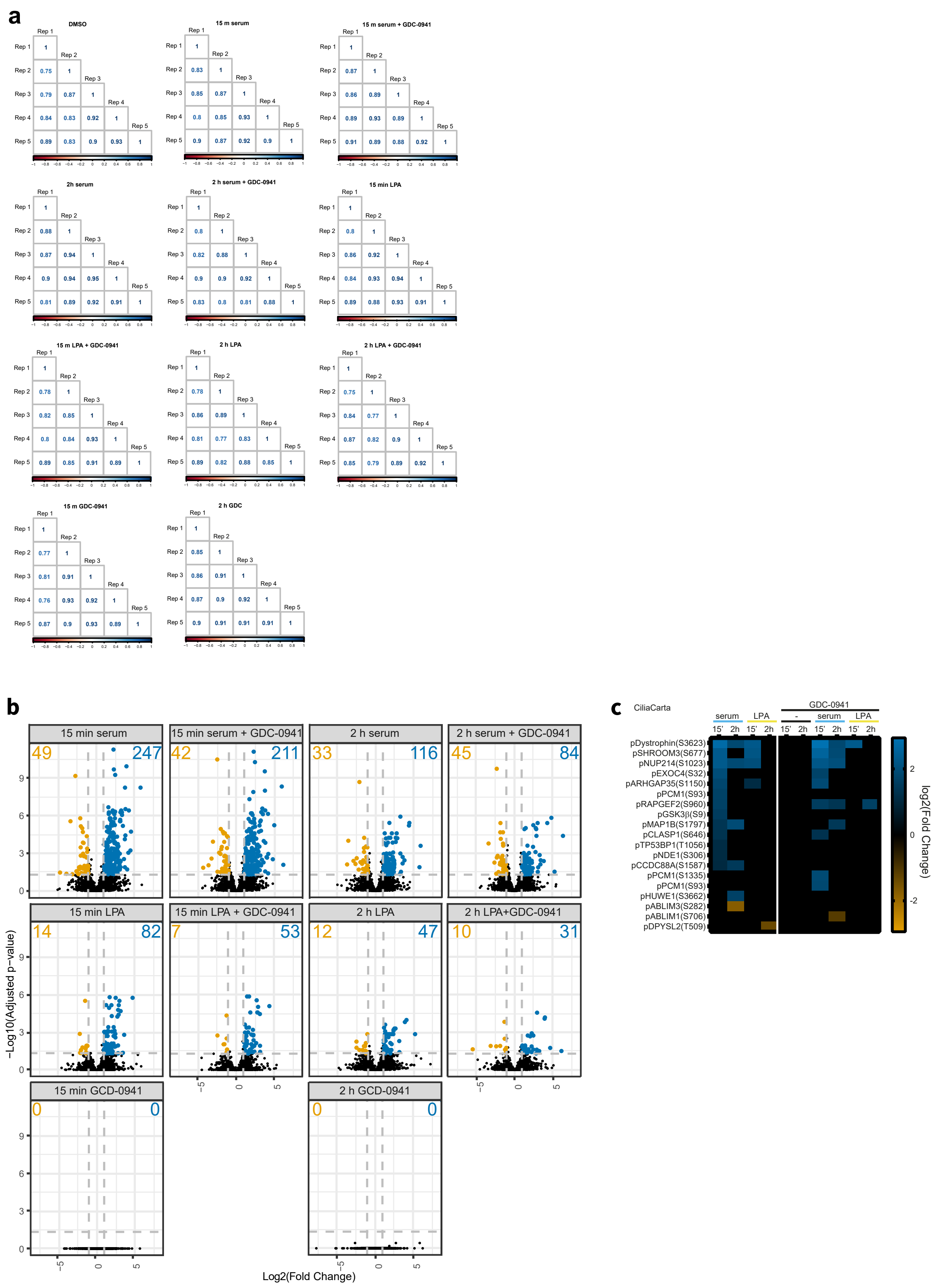

Supplementary Fig. 2 | Set-up of phosphoproteomic experiment 1 and control data. a, Multi-scatter plot of the Log2(intensity) of signals obtained from each replicate against the Log2(intensity) of the same sample from all other replicates. Numbers indicate the Pearson correlation coefficient for each pair, n=5 independent experiments. **b,** Phosphoproteomic analysis of hTERT-RPE1 cells serum-starved for 48 h and stimulated with 10% serum or 2 μM LPA ± 0.5 μM GDC-0941 for 15 min or 2 h. Volcano plot of phosphosites differentially regulated by serum or LPA ± GDC-0941 relative to DMSO-treated cells. Numbers in the top corners of the plot indicate the number of phosphosites significantly up- or down-regulated in each condition relative to DMSO, n=5 independent experiments (p-value calculated using the group comparison function within MSstats and adjusted to control for multiple comparisons using the Benjamini-Hochberg procedure, n=5 independent experiments). **c,** Heatmap displaying phosphosites from cilia-associated proteins (as defined by CiliaCarta) regulated by 15 min or 2 h serum or 2 μM LPA stimulation ± 0.5 μM GDC-0941 in 48 h serum-starved hTERT-RPE1 cells relative to DMSO, n=5 independent experiments.

Supplementary Figure 3

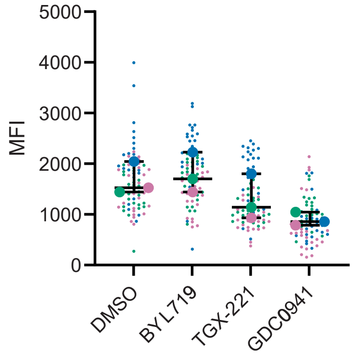

Supplementary Fig. 3 | Basal ciliary PIP_3 is produced by PI3Kβ in hTERT-RPE1 cells hTERT-RPE1 cells were serum-starved for 48 h and treated with 0.25 μM BYL719, 0.5 μM TGX-221, 0.5 μM GDC-0941 or DMSO for 1 h. Cells were fixed, stained with ARL13B and PIP_3 antibodies and DAPI and imaged by confocal microscopy. To measure the ciliary PIP_3 MFI, for each cilium, a box of standardised size was placed at the base of the ARL13B demarked axoneme centred around the highest intensity PIP_3 pixel and the MFI within the box measured. The data from Fig. 4a are reproduced here as a SuperPlot, bars represent median $\pm$ 95% confidence interval, n>75 cells per condition from 3 independent experiments.

Supplementary Figure 4

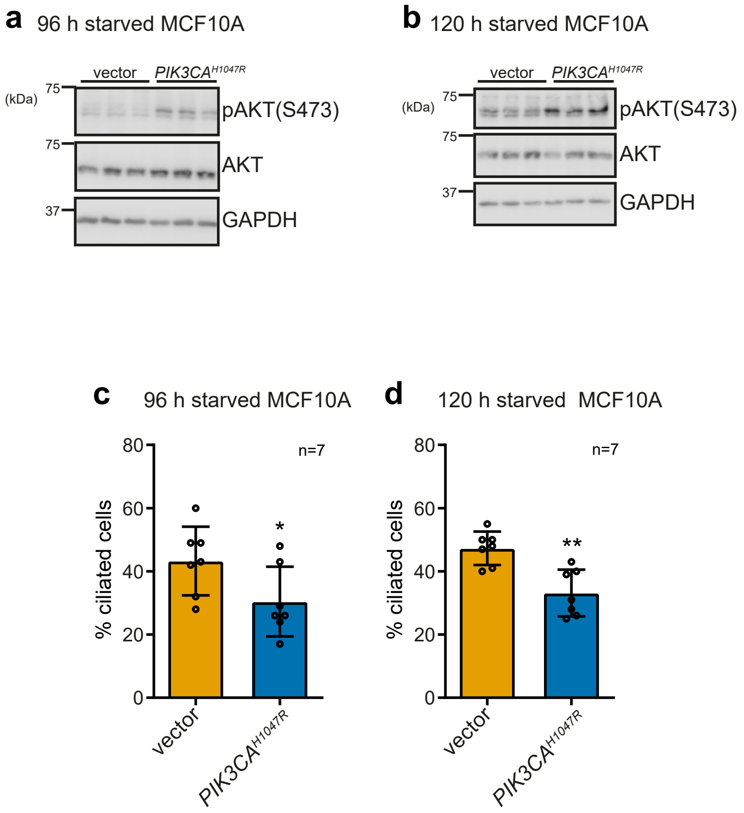

Supplementary Fig. 4 | Validation of *PIK3CA*^{H1047R} MCF10A cells. a-b, Vector and *PIK3CA*^{H1047R} MCF10A cells were serum-starved for (**a**) 96 h or (**b**) 120 h with 0.5 μg/ml doxycycline, lysed and immunoblotted with pAKT(S473), AKT and GAPDH antibodies, blots representative of n=3 independent experiments. **c-d,** Vector and *PIK3CA*^{H1047R} MCF10A cells were serum-starved for 96 h (**c**) or 120 h (**d**) with 0.5 μg/ml doxycycline, fixed, stained with ARL13B and pericentrin antibodies and DAPI and the percentage of ciliated cells scored, bars indicate mean ± SD, 100 cells scored per condition per independent experiment, n=7 independent experiments, *p<0.05, **p<0.01, (Student's t-test (**c**) p=0.0483, (**d**) p=0.0014). Source data are provided as a Source Data file.

Supplementary Figure 5

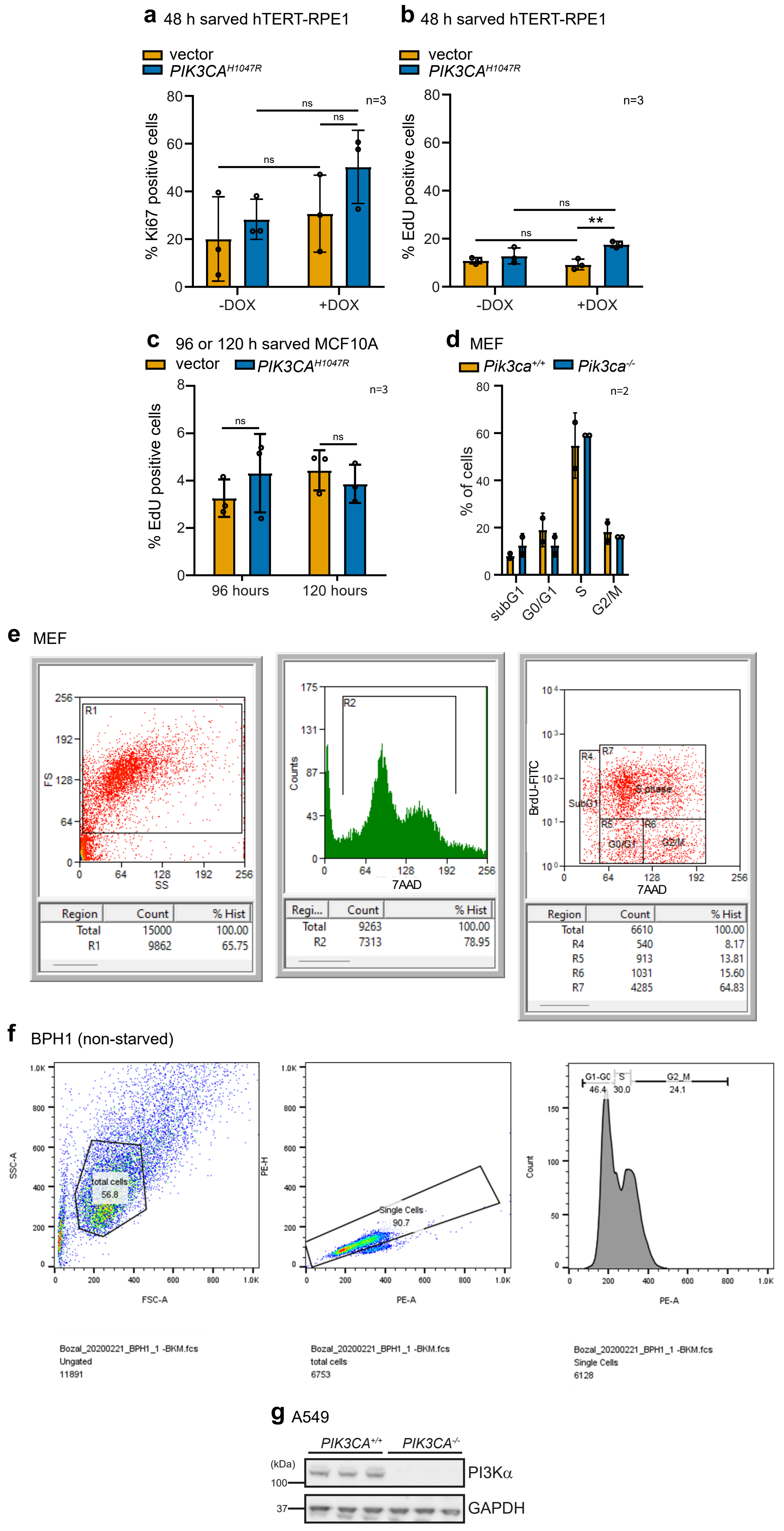

Supplementary Fig. 5 | No overt cell cycle perturbation by PI3Kα activation under ciliated conditions. a-b, Vector and *PIK3CA*^{H1047R} hTERT-RPE1 cells were with serum-starved for 48 h ± 0.5 μg/ml doxycycline (DOX) and (**b**) labelled with EdU for 4 h. Cells were fixed, stained with Ki67 antibodies (**a**) or for EdU (**b**) and DAPI and imaged by confocal microscopy. The percentage of (**a**) Ki67 or (**b**) EdU-positive cells was scored, bars indicate mean ± SD, n=3 independent experiments, ns not significant, **p<0.01 (Two-way ANOVA (**a**) interaction, p=0.5251, row factor p=0.0940, column factor p=0.1430, (**b**) interaction p=0.0364, row factor p=0.2483, column factor p=0.0037). **c,** Vector and *PIK3CA*^{H1047R} MCF10A cells were serum-starved for 96 or 120 h with 0.5 μg/ml doxycycline and labelled with EdU for 4 h. Cells were fixed, stained for EdU and DAPI and imaged by confocal microscopy. The percentage of EdU-positive cells was scored, bars indicate mean ± SD, n=3 independent experiments, ns, not significant (Two-way ANOVA interaction p=0.2333, row factor p=0.5815, column factor p=0.7125). **d,** Cell cycle distribution of *Pik3ca*$^{+/+}$ and *Pik3ca*$^{-/-}$ MEFs assessed by flow cytometry analysis of BrdU incorporation, (d) n=2 independent experiments, (e) representative grating strategy for flow cytometry. **f,** Representative gating strategy for flow cytometry analysis presented in Fig. 5g. **g,** *Pik3ca*$^{+/+}$ and *Pik3ca*$^{-/-}$ A549 cells were immunoblotted with PI3Kα and GAPDH antibodies, each lane represents an independent experiment. Source data are provided as a Source Data file.

Supplementary Figure 6

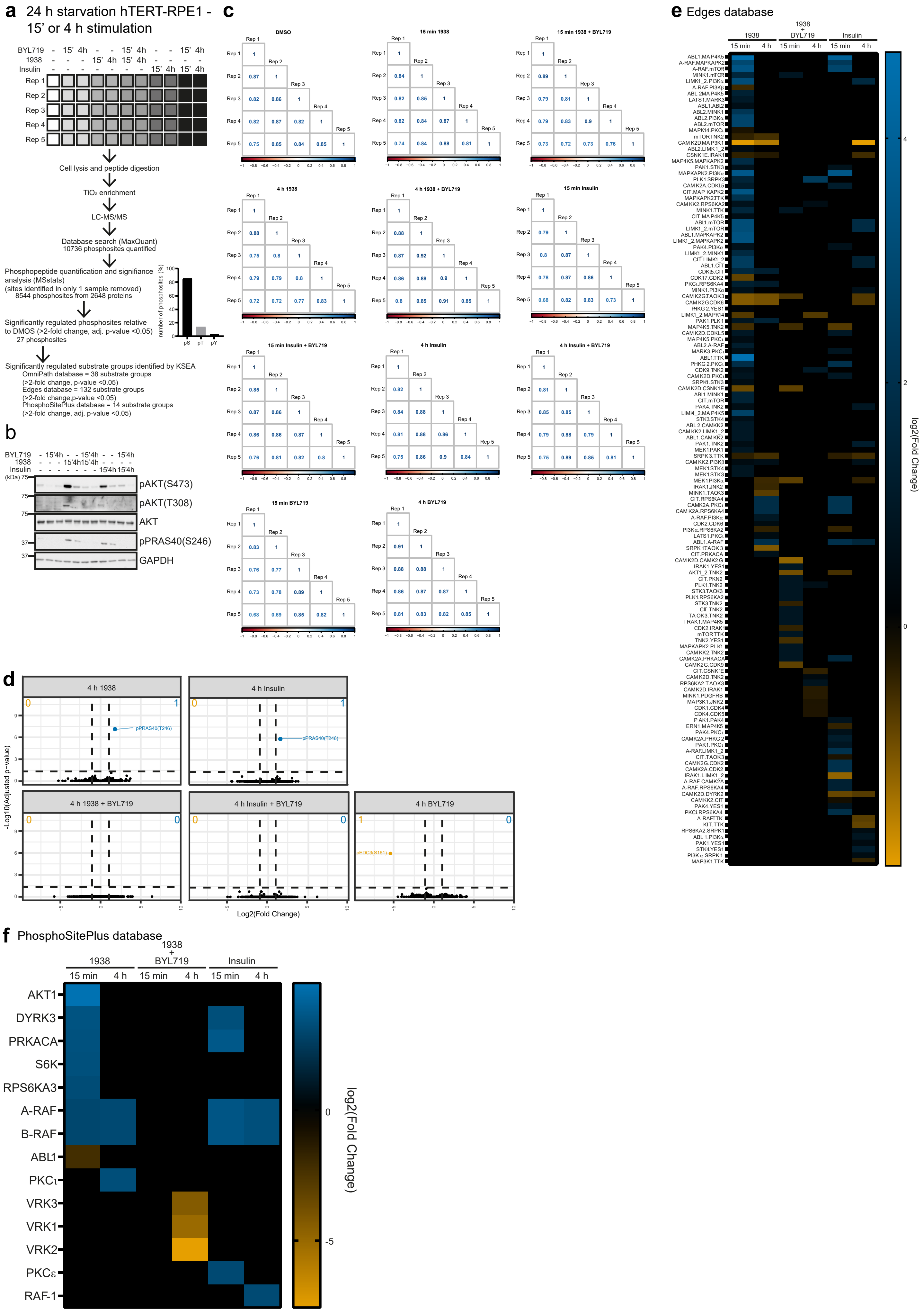

Supplementary Fig. 6 | Set-up of phosphoproteomic experiment 2 and control data. a, Experimental design and workflow of phosphoproteomic experiment. hTERT-RPE1 cells were serum-starved for 24 h and stimulated with 5 μM 1938 or 100 nM insulin ± 0.25 μM BYL719 for 15 min or 4h and processed for phosphoproteomic analysis. 8544 phosphosites from 2648 proteins were analysed by MSstats, 27 phosphosites were significantly regulated which were for KSEA (n=5 independent experiments). **b,** hTERT-RPE1 cells were serum-starved for 24 h and stimulated with 5 μM 1938 or 100 nM insulin ± 0.25 μM BYL719 for 15 min or 4 h. Cells were lysed and immunoblotted with pAKT(S473), pAKT(T308), AKT, pPRAS40(S246) and GAPDH antibodies, blots representative of n=3 independent experiments. **c,** Multi-scatter plot of the Log2(intensity) of signals obtained from each replicate against the Log2(intensity) of the same sample from all other replicates. Numbers indicate the Pearson correlation coefficient for each pair, n=5 independent experiments. **d,** Phosphoproteomic analysis of hTERT-RPE1 cells serum-starved for 24 h and stimulated with 5 μM 1938 or 100 nM insulin ± 0.25 μM BYL719 for 4 h (n=5 independent experiments). Volcano plot of phosphosites differentially regulated by 1938 or insulin ± BYL719 relative to DMSO-treated cells. Numbers in the top corners of the plot indicate the number of phosphosites significantly up- or down-regulated in each condition relative to DMSO (p-value calculated using the group comparison function within MSstats and adjusted to control for multiple comparisons using the Benjamini-Hochberg procedure, n=5 independent experiments). **e-f,** Heatmap displaying KSEA (using the (**e**) Edges or (**f**) PhosphoSitePlus database) of kinases for which the substrate groups are differentially regulated by 15 min or 4 h 1938 or Insulin stimulation ± BYL719 in 24 h serum-starved hTERT-RPE1 cells relative to DMSO, n=5 independent experiments. As few phosphosites were altered by 1938 or insulin treatment, for KSEA using the Edges database, kinases for which the raw p-values (using the Kolmogorov–Smirnov test) relative to DMSO control were less than 0.05 were considered significantly regulated. For KSEA using the PhosphoSitePlus database, adjusted p-values (using the Kolmogorov–Smirnov test followed by Benjamini-Hochberg principle (5% FDR)) relative to DMSO control less than 0.05 were considered statistically significantly.

Supplementary Figure 7

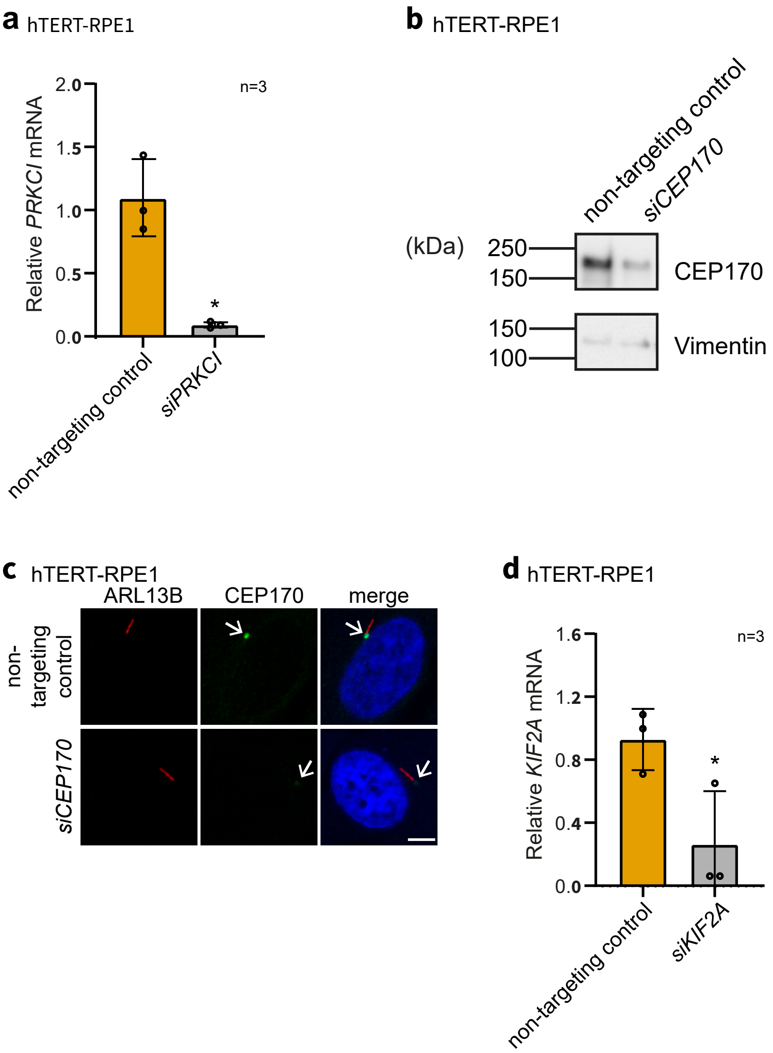

Supplementary Fig. 7 | Validation of siRNA knockdown models. a, hTERT-RPE1 cells were transfected with non-targeted control or *PRKCI* siRNA and incubated for 72 h, lysed and *PRKCI* mRNA levels measured relative to *GAPDH* mRNA via qRT-PCR, bars indicate mean ± SD, n=3 independent experiments, *p<0.05 (Student's t-test, (A) p=0.0289). **b,** hTERT-RPE1 cells were transfected with non-targeted control or *CEP170* siRNA and incubated for 48 h, lysed and immunoblotted with CEP170 and GAPDH antibodies, blots representative of n=3 independent experiments. Sample processing controls were run on a different gel in parallel with an equal amount of protein loaded per well. **c,** hTERT-RPE1 cells were transfected with non-targeted control or *CEP170* siRNA and incubated for 48 h. Cells were fixed, stained with ARL13B and CEP170 antibodies and DAPI and imaged by confocal microscopy, arrows indicate CEP170 signals, bar indicates 5 μm, representative of n=3 transfections. **d,** hTERT-RPE1 cells were transfected with non-targeted control or *KIF2A* siRNA and incubated for 72 h, lysed and *KIF2A* mRNA levels measured relative to *GAPDH* mRNA via qRT-PCR, bars indicate mean ± SD, n=3 independent experiments, *p<0.05 (Student's t-test p=0.0413). Source data are provided as a Source Data file.

Supplementary Table 1: Genotypes of embryos observed from heterozygous *CreDel* mice mated with heterozygous *Pik3ca*^{H1047R} mice.

	Pik3CA$^{+/+}$	*Pik3ca*$^{+/+}$;*CreDel*	*Pik3ca*$^{H1047R/+}$	*Pik3ca*$^{H1047R/+}$;*CreDel*	total
E8.5	4	6	3	7	20
E9.0	9	8	8	15	40
E9.5	68	59	65	48	240
total	81	73	76	70	300

Supplementary Table 2: Phenotypes of E9.5 *Pik3ca*^{H1047R} embryos.

Genotype	Phenotype (%, embryos with phenotype/number of embryos)				
	small size	shorting of posterior trunk	craniofacial deficiency	reduced somites	defective turning
control	0%, 0/4	0%, 0/4	0%, 0/4	0%, 0/4	0%, 0/4
Pik3ca^{H1047R}	83%, 5/6	83%, 5/6	100%, 6/6	83%, 5/6	33%, 2/6

Supplementary Table 3: Tukey's post-hoc analysis for Fig. 7h. hTERT-RPE1 cells were serum-starved for 48 h, pre-treated with 0.25 μM BYL719, 0.5 μM TGX-221, 0.5 μM GDC-0941, 5 μM GSK2334470, 2 μM MK2206, 2 μM 229 or DMSO for 1 h and then stimulated with serum in the presence of inhibitors for 24 h. Left, cells were fixed, stained with ARL13B and pericentrin antibodies and DAPI and the percentage of ciliated cells scored (one-way ANOVA, $p<0.0001$).

Tukey' multiple comparisons	Adjusted p-value	Summary
Starve vs. DMSO	2.192×10^{-10}	****
Starve vs. BYL719	2.932×10^{-6}	****
Starve vs. TGX-221	9.728×10^{-6}	****
Starve vs. GDC0941	9.211×10^{-5}	****
Starve vs. GSK2334470	0.0532	ns
Starve vs. MK2206	0.0005	***
Starve vs.229	0.0007	***
DMSO vs. BYL719	0.0191	*
DMSO vs. TGX-221	0.0063	**
DMSO vs. GDC0941	0.0007	***
DMSO vs. GSK2334470	8.957×10^{-7}	****
DMSO vs. MK2206	1.302×10^{-4}	***
DMSO vs. 229	9.211×10^{-5}	****
BYL719 vs. TGX-221	0.9999	ns
BYL719 vs. GDC0941	0.9223	ns
BYL719 vs. GSK2334470	0.0222	*
BYL719 vs. MK2206	0.6127	ns
BYL719 vs. 229	0.5354	ns
TGX-221 vs. GDC0941	0.9926	ns
TGX-221 vs. GSK2334470	0.0611	ns
TGX-221 vs. MK2206	0.8532	ns
TGX-221 vs. 229	0.7936	ns
GDC0941 vs. GSK2334470	0.2925	ns
GDC0941 vs. MK2206	0.9985	ns
GDC0941 vs. 229	0.9954	ns
GSK2334470 vs. MK2206	0.6511	ns
GSK2334470 vs. 229	0.7254	ns
MK2206 vs. 229	0.99999997	ns

Original immunoblots

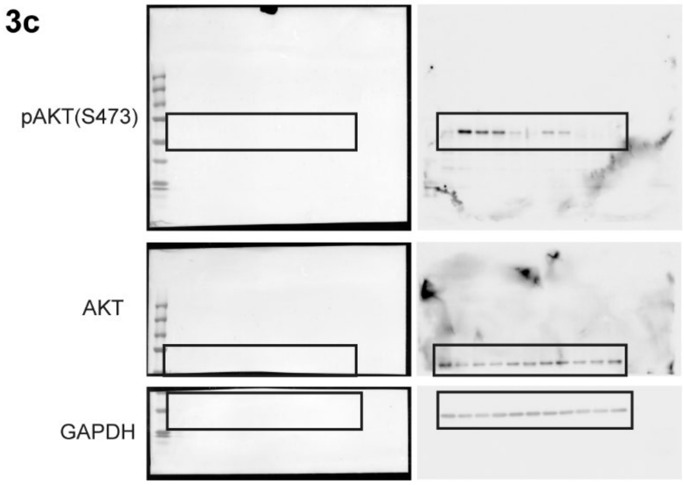

Original immunoblots

5e

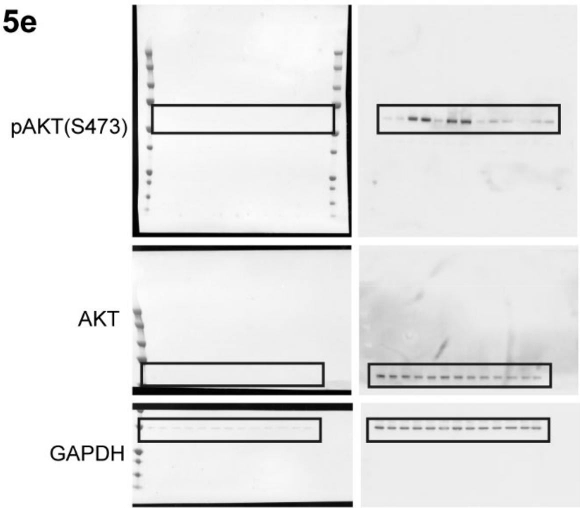

Original immunoblots

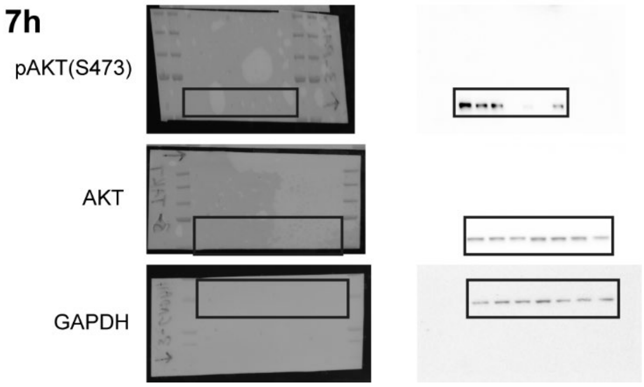

Original immunoblots

Supplementary 1c

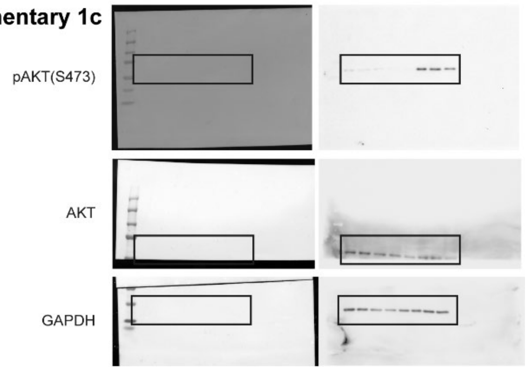

Original immunoblots

Supplementary 4a

pAKT(S473) AKT GAPDH

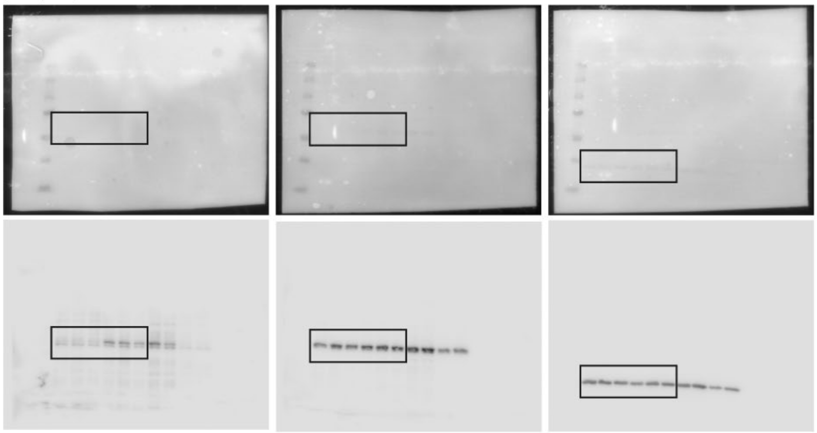

Supplementary 4b

pAKT(S473) AKT

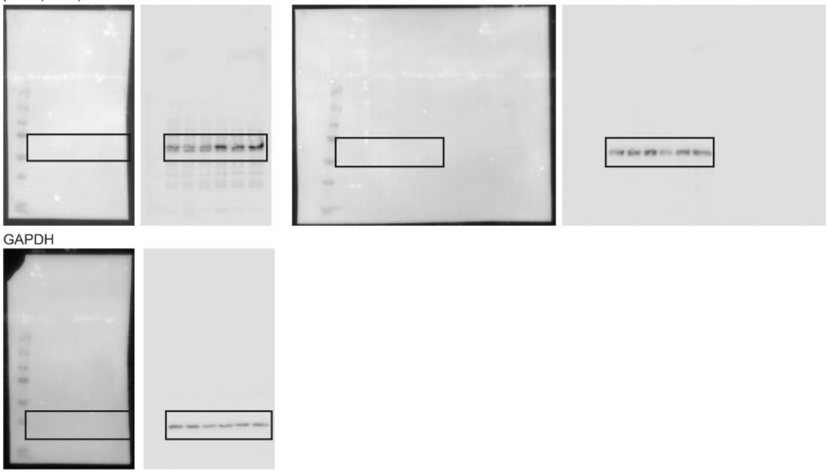

Original immunoblots

Supplementary 5g

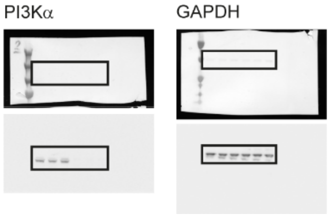

Original immunoblots

Supplementary 6b

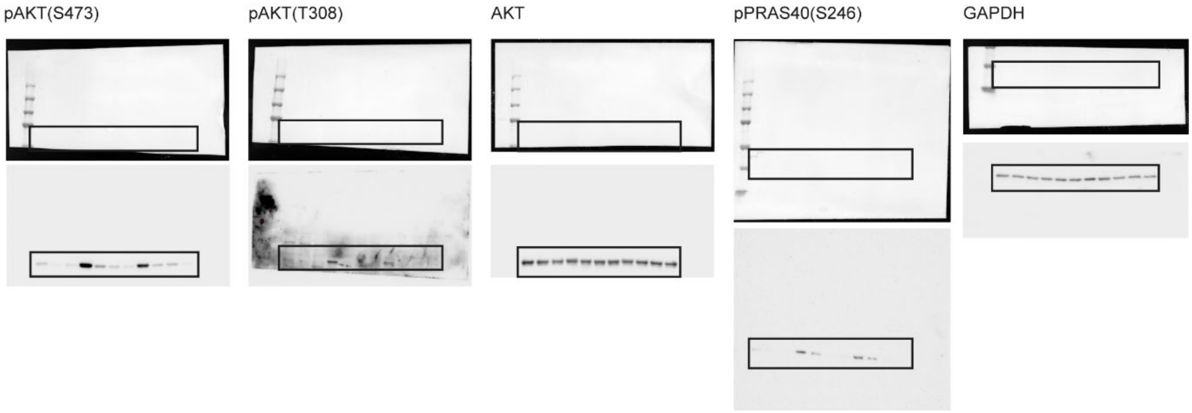

Original immunoblots

Supplementary 7b

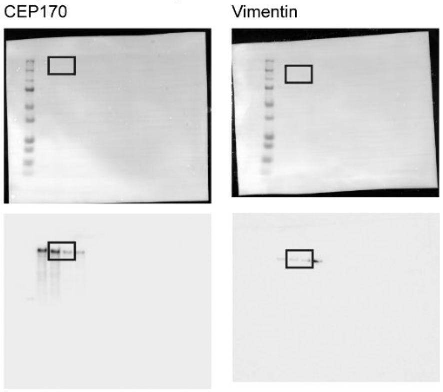

Supplement: Supplementary file 1 — Supplementary Information [file 41467_2024_51354_MOESM1_ESM.pdf]
