# Supplementary material for: A class I PI3K signalling network regulates primary cilia disassembly in normal physiology and disease
Source: Nat Commun. 2024 Aug 21;15:7181. doi: 10.1038/s41467-024-51354-1 (PMC11339396; doi:10.1038/s41467-024-51354-1)
Supplement: Supplementary file 3 — Description of Additional Supplementary Information [file 41467_2024_51354_MOESM3_ESM.docx]

**Description of Additional Supplementary Files**

File Name: Supplementary Data 1

Description: Comparison of Pik3caH1047R phenotypes with cilia, Hedgehog and Wnt signalling mutants.

File Name: Supplementary Data 2

Description: Phosphoproteomics analysis of serum- and LPA-stimulated hTERT-RPE1 cells.

File Name: Supplementary Data 3

Description: Phosphoproteomics analysis of 1938- and insulin-stimulated hTERT-RPE1 cells.
