# Supplementary material for: A class I PI3K signalling network regulates primary cilia disassembly in normal physiology and disease
Source: Nat Commun. 2024 Aug 21;15:7181. doi: 10.1038/s41467-024-51354-1 (PMC11339396; doi:10.1038/s41467-024-51354-1)
Supplement: Supplementary file 7 — Reporting Summary [file 41467_2024_51354_MOESM7_ESM.pdf]

nature portfolio

Corresponding author(s): Bart Vanhaesebroeck
Sarah Conduit

Last updated by author(s): 2024/07/25

Reporting Summary

Nature Portfolio wishes to improve the reproducibility of the work that we publish. This form provides structure for consistency and transparency in reporting. For further information on Nature Portfolio policies, see our Editorial Policies and the Editorial Policy Checklist.

Statistics

For all statistical analyses, confirm that the following items are present in the figure legend, table legend, main text, or Methods section.

n/a	Confirmed	
[ ]	[x]	The exact sample size (*n*) for each experimental group/condition, given as a discrete number and unit of measurement
[ ]	[x]	A statement on whether measurements were taken from distinct samples or whether the same sample was measured repeatedly
[ ]	[x]	The statistical test(s) used AND whether they are one- or two-sided<br>*Only common tests should be described solely by name; describe more complex techniques in the Methods section.*
[x]	[ ]	A description of all covariates tested
[ ]	[x]	A description of any assumptions or corrections, such as tests of normality and adjustment for multiple comparisons
[ ]	[x]	A full description of the statistical parameters including central tendency (e.g. means) or other basic estimates (e.g. regression coefficient) AND variation (e.g. standard deviation) or associated estimates of uncertainty (e.g. confidence intervals)
[ ]	[x]	For null hypothesis testing, the test statistic (e.g. *F*, *t*, *r*) with confidence intervals, effect sizes, degrees of freedom and *P* value noted<br>*Give P values as exact values whenever suitable.*
[x]	[ ]	For Bayesian analysis, information on the choice of priors and Markov chain Monte Carlo settings
[x]	[ ]	For hierarchical and complex designs, identification of the appropriate level for tests and full reporting of outcomes
[ ]	[x]	Estimates of effect sizes (e.g. Cohen's *d*, Pearson's *r*), indicating how they were calculated

Our web collection on statistics for biologists contains articles on many of the points above.

Software and code

Policy information about availability of computer code

Data collection	Image acquisition software, Zen Black (version 14.0.0.0), Zen 3.3 Blue edition, Leica Application Suite (version 4.0.0).
Data analysis	Image analysis software, Fiji ImageJ, Imaris (version 10.1.0). Phosphoproteomics raw data analysis software, MaxQuant (version 1.6.17). Analysis of quantified phosphopeptides software, MSstats(version 3.20.0, run through RStudio (version 1.3.1093, R version 4.0.3)). KSEA scripts are available at github/CutillasLab. Flow analysis was performed using FlowJo v10.10 and Summit V4.0 (DakoCytomation). Statistical analysis software, GraphPad Prism 8.0 or 10.1.2.

For manuscripts utilizing custom algorithms or software that are central to the research but not yet described in published literature, software must be made available to editors and reviewers. We strongly encourage code deposition in a community repository (e.g. GitHub). See the Nature Portfolio guidelines for submitting code & software for further information.

Data

Policy information about availability of data

All manuscripts must include a data availability statement. This statement should provide the following information, where applicable:

- Accession codes, unique identifiers, or web links for publicly available datasets
- A description of any restrictions on data availability
- For clinical datasets or third party data, please ensure that the statement adheres to our policy

Mass spectrometry data (raw data, processed data and scripts) have been deposited to the ProteomeXchange Consortium via the PRIDE partner repository (Perez-Riverol, 2019), with the dataset identifier PXD046456. Source data are provided with this paper. The other data that support the findings in this study are available

from the corresponding author upon request.

Research involving human participants, their data, or biological material

Policy information about studies with human participants or human data. See also policy information about sex, gender (identity/presentation), and sexual orientation and race, ethnicity and racism.

Reporting on sex and gender	Human participants were not used in this study.
Reporting on race, ethnicity, or other socially relevant groupings	As per box 1 above, race, ethnicity or other social groupings are not relevant to this study which did not use human participants.
Population characteristics	As per box 1 above, population characteristics are not relevant to this study which did not use human participants.
Recruitment	As per box 1 above, recruitment is not relevant to this study which did not use human participants.
Ethics oversight	As per box 1 above human ethics oversight is not relevant to this study which did not use human participants.

Note that full information on the approval of the study protocol must also be provided in the manuscript.

Field-specific reporting

Please select the one below that is the best fit for your research. If you are not sure, read the appropriate sections before making your selection.

[x] Life sciences   [ ] Behavioural & social sciences   [ ] Ecological, evolutionary & environmental sciences

For a reference copy of the document with all sections, see nature.com/documents/nr-reporting-summary-flat.pdf

Life sciences study design

All studies must disclose on these points even when the disclosure is negative.

Sample size	Samples sizes were chosen based on our and our collaborators prior experience and the standards used in the field.
Data exclusions	No data were excluded form the analysis.
Replication	All experiments were performed for at least n=3 biological replicates as indicated in the figure legends.
Randomization	Mice were allocated to groups based on their genotype. Randomization is not relevant to the cell biology experiment presented here.
Blinding	Data from mouse embryos was collected prior to genotyping, therefore investigators were blinded. Investigators were not blinded for cell biology experiments.

Reporting for specific materials, systems and methods

We require information from authors about some types of materials, experimental systems and methods used in many studies. Here, indicate whether each material, system or method listed is relevant to your study. If you are not sure if a list item applies to your research, read the appropriate section before selecting a response.

Materials & experimental systems

n/a	Involved in the study	
[ ]	[x]	Antibodies
[ ]	[x]	Eukaryotic cell lines
[x]	[ ]	Palaeontology and archaeology
[ ]	[x]	Animals and other organisms
[x]	[ ]	Clinical data
[x]	[ ]	Dual use research of concern
[x]	[ ]	Plants

Methods

n/a	Involved in the study	
[x]	[ ]	ChIP-seq
[ ]	[x]	Flow cytometry
[x]	[ ]	MRI-based neuroimaging

Antibodies

Antibodies used	pAKT(S473), Cell Signalling Technologies (#9271)<br>pAKT(T308), Cell Signalling Technologies (#9275)<br>AKT, Cell Signalling Technologies (#9272)<br>PI3Ka, Cell Signalling Technologies (#4249)

	pPRAS40(S246), Cell Signalling Technologies (#2640)<br>Ki67, Cell Signalling Technologies (Alexa Fluor 488 Conjugate, #11882)<br>pS6RP(Ser240/244), Cell Signalling Technologies (#5364)<br>ARL13B, Abcam (ab136648)<br>AlexaFluor647-conjugated anti-ERG, Abcam (ab196149)<br>GAPDH, Abcam (ab8245)<br>Pericentrin, Abcam (ab4448)<br>ARL13B, Proteintech (17711-1-AP)<br>CEP170, Sigma-Aldrich (HPA042151)<br>γ-tubulin, Sigma-Aldrich (T5326)<br>Acetylated α-tubulin, Sigma-Aldrich (T7451)<br>HA, BioLegend (HA.11)<br>PI(3,4)P2, Echelon Biosciences (Z-P034)<br>PI(3,4,5)P3, Echelon Biosciences (Z-P345b)<br>SMO, Santa Cruz Biotechnology (sc-166685)<br>Alexa-Fluor-488/568/594/647-conjugated mouse and rabbit secondary antibodies, ThermoFisher Scientific<br>AlexaFluor568-conjugated isolectin GS-B4, ThermoFisher Scientific (I21412)<br>HRP-conjugated mouse (NXA931V) and rabbit (NA934V) secondary antibodies, Cytiva
Validation	All antibodies have been validated either by the company or ourselves.

Eukaryotic cell lines

Policy information about cell lines and Sex and Gender in Research

Cell line source(s)	hTERT-RPE1 cells were purchased from ATCC (Manassas, VA, USA; CRL-4000)<br>HEK293 (LentiX) cells were purchased from Clontech (Mountain View, CA, USA, NC9834960)<br>MCF10A cells were purchased from ATCC (CRL-10317)<br>A549 cells were purchased from ATCC (CCL-185)<br>BPH1 cells were purchased from DSMZ (ACC 143, ref.14602)<br>Immortalised Pik3ca+/+ and Pik3ca-/- MEFs were generated and described previously (Foukas et al, PNAS 2010)
Authentication	The authenticity of hTERT-RPE1 cells is tested by ATCC using short tandem repeat analysis, immunocytochemistry for pan-cytokeratin and flow cytometry for Ep-16 expression. Bulk frozen stocks were prepared immediately following receipt and used within 2 months of defrosting.
Mycoplasma contamination	All cell lines were tested regularly for mycoplasma and confirmed to be negative.
Commonly misidentified lines<br>(See ICLAC register)	No commonly misidentified cell lines were used in this study.

Animals and other research organisms

Policy information about studies involving animals; ARRIVE guidelines recommended for reporting animal research, and Sex and Gender in Research

Laboratory animals	All mice were on the C57BL/6 background. Mouse embryo age is stated in the relavent figure legend for each experiment.
Wild animals	The study did not involve wild animals.
Reporting on sex	Male and female embryos were used for analysis. Sex based analysis was not performed as embryos were only analysed up to E10.5 which is the bipotential stage during which the developing male and female reproductive systems are identical.
Field-collected samples	The study did not involve samples collected from the field.
Ethics oversight	Mouse studies were performed according to UK The Animals (Scientific Procedures) Act 1986 Amendment Regulations 2012 (approved by the Animal Welfare and Ethical Review Body (AWERB), P434BB714 and PP5281579) and the Catalan Ministry of Agriculture, Livestock, Fisheries and Food guidelines (protocols approved by CEEA Ethics Committees; animal Use Protocol number 9725).

Note that full information on the approval of the study protocol must also be provided in the manuscript.

Plants

Seed stocks	No plants were used in this study.
Novel plant genotypes	As per box 1 above, novel plant genotypes are not relevant to this study which did not use plants.
Authentication	As per box 1 above, plant authentication is not relevant to this study which did not use plants.

Flow Cytometry

Plots

Confirm that:

- [x] The axis labels state the marker and fluorochrome used (e.g. CD4-FITC).
- [x] The axis scales are clearly visible. Include numbers along axes only for bottom left plot of group (a 'group' is an analysis of identical markers).
- [x] All plots are contour plots with outliers or pseudocolor plots.
- [x] A numerical value for number of cells or percentage (with statistics) is provided.

Methodology

Sample preparation	For cell cycle analysis of BPH1, cells were collected in PBS, fixed in 4% PFA for 15 min at room temperature and permeabilized with 0.2% Triton X-100 in PBS for 30 min at room temperature. Then, pelleted cells were stained with propidium iodide solution (2% PI and 0.1 mg/ml RNAse A in PBS) for 30 min at 37ºC and analysed by FACS (BD Biosciences).<br>For cell cycle analysis of MEFs, MEFs were treated as described in the Methods. Staining of samples was performed with a BrdU Flow Kit according to the manufacturer (BD Pharmigen) instructions.
Instrument	For cell cycle analysis of BPH1 a BD Biosciences Instrument was used. For cell cycle analysis of MEFs an CyAn ADP (DakoCytomation) was used.
Software	For cell cycle analysis of BPH1 FlowJo v10.10 was used. For cell cycle analysis of MEFs Summit V4.0 (DakoCytomation) was used.
Cell population abundance	Cell population abundance does not apply to these experiments.
Gating strategy	For cell cycle analysis of BPH1. Forward versus side scatter density plots (FSC vs SSC) gating was used identify cell population of interest based on size and granularity and to exclude debris . A forward scatter height (FSC-H) vs. forward scatter area (FSC-A) density plot was then used to exclude doublets. A single parameter histogram was next used to further identify cell population.<br>For cell cycle analysis for MEFs. An initial gate was set in the FS/SS plot to exclude cell debris. A second gate was set to select cells with 2n and 4n DNA to exclude doublets and clumps. Cells with fluorescence intensity higher than 10^1 were considered BrdU positive.

- [x] Tick this box to confirm that a figure exemplifying the gating strategy is provided in the Supplementary Information.
